# Cost-effectiveness of nurse-led multifactorial care to prevent or postpone new disabilities in community-living older people: Results of a cluster randomized trial

**DOI:** 10.1371/journal.pone.0175272

**Published:** 2017-04-17

**Authors:** Jacqueline J. Suijker, Janet L. MacNeil-Vroomen, Marjon van Rijn, Bianca M. Buurman, Sophia E. de Rooij, Eric P. Moll van Charante, Judith E. Bosmans

**Affiliations:** 1 Department of General Practice, Academic Medical Center, Amsterdam, The Netherlands; 2 Department of Internal Medicine, Section of Geriatric Medicine, Academic Medical Center, Amsterdam, The Netherlands; 3 University Center for Geriatric Medicine, University Medical Center Groningen, Groningen, The Netherlands; 4 Department of Health Sciences and EMGO Institute for Health and Care Research, Vrije Universiteit Amsterdam, Amsterdam, The Netherlands; TNO, NETHERLANDS

## Abstract

**Objective:**

To evaluate the cost-effectiveness of nurse-led multifactorial care to prevent or postpone new disabilities in community-living older people in comparison with usual care.

**Methods:**

We conducted cost-effectiveness and cost-utility analyses alongside a cluster randomized trial with one-year follow-up. Participants were aged ≥ 70 years and at increased risk of functional decline. Participants in the intervention group (n = 1209) received a comprehensive geriatric assessment and individually tailored multifactorial interventions coordinated by a community-care registered nurse with multiple follow-up visits. The control group (n = 1074) received usual care. Costs were assessed from a healthcare perspective. Outcome measures included disability (modified Katz-Activities of Daily Living (ADL) index score), and quality-adjusted life-years (QALYs). Statistical uncertainty surrounding Incremental Cost-Effectiveness Ratios (ICERs) was estimated using bootstrapped bivariate regression models while adjusting for confounders.

**Results:**

There were no statistically significant differences in Katz-ADL index score and QALYs between the two groups. Total mean costs were significantly higher in the intervention group (EUR 6518 (SE 472) compared with usual care (EUR 5214 (SE 338); adjusted mean difference €1457 (95% CI: 572; 2537). Cost-effectiveness acceptability curves showed that the maximum probability of the intervention being cost-effective was 0.14 at a willingness to pay (WTP) of EUR 50,000 per one point improvement on the Katz-ADL index score and 0.04 at a WTP of EUR 50,000 per QALY gained.

**Conclusion:**

The current intervention was not cost-effective compared to usual care to prevent or postpone new disabilities over a one-year period. Based on these findings, implementation of the evaluated multifactorial nurse-led care model is not to be recommended.

## Introduction

With the aging of the population and the associated increase in multimorbidity, the prevention of (new) disability in community-living older persons has received considerable attention.[1] Disability is defined as difficulty or dependence in performing (instrumental) daily activities essential for independent living [2], while the occurrence of new physical disabilities is often referred to as functional decline.[3] Functional decline in older people is associated with loss of quality of life,[4] loss of independence,[5] and strains on social and economic resources.[6] It has been suggested that proactive, integrated care provision for community-living older people with complex care needs may postpone new disabilities, support independent living, and curtail health and social costs by preventing, delaying, or reducing hospitalizations and nursing home admissions.[2, 7–12]

Although some recent studies concluded that multifactorial interventions were not cost-effective compared to usual care,[13–16] other studies described that they were cost-effective at high willingness-to-pay ratios.[17–19] Despite conflicting evidence on the (cost-) effectiveness of multifactorial interventions to prevent or postpone new disabilities as shown in a number of reviews, [20–27] different proactive strategies are already part of national policy of elderly care in several Western countries.[28]

Over the last two decades, in the Netherlands, there has been an increasing task delegation towards registered nurses (RN) working in general practice, especially in the care for patients with chronic conditions.[29] This has become fertile soil to implement multifactorial nurse-led interventions to prevent new disabilities in older people. In 2008, the Dutch government launched the National Care for the Elderly Programme (NCEP) stimulating innovative healthcare projects focused on older people with multifactorial care needs to promote physical, mental and social health and well-being.[30] We designed an intervention to prevent or postpone new disabilities targeting a community-living primary care population, at increased risk of functional decline, and offered them geriatric interventions based on current evidence or guidelines, patient-centered care, and nurse-led care coordination.[20, 23, 25, 31, 32] All these components were designed based on reviews and meta-analyses of interventions with a beneficial effect on overall functioning. The aim of this study was to investigate the cost-effectiveness of a multifactorial nurse-led care to prevent or postpone new disabilities in community-living older people at increased risk of functional decline compared to usual care from a healthcare perspective alongside a cluster randomized trial.

## Methods

### Design

We conducted a cost-effectiveness analysis (CEA) and a cost-utility analysis (CUA) from a healthcare perspective alongside a cluster randomized trial with a one-year follow-up in the Netherlands. The trial was registered at Trial Registration NTR2653 http://www.trialregister.nl). The study protocol was approved by the Medical Ethics Committee of the Academic Medical Center, University of Amsterdam, The Netherlands (protocol ID MEC10/182) and all eligible participants signed a written informed postponed consent before inclusion. We provide a summary of the materials and methods in the current article. A more detailed description of the materials and methods is published in the study protocol.[31] The results of the trial are published elsewhere.[32]

### Setting and participants

The cluster randomized trial was conducted between December 2010 and May 2014, in the north-west of the Netherlands.[32] Twenty-four general practices who had not implemented nurse-led integrated care for community-living older people, participated. All participating general practitioners (GP) invited their patients aged 70 years and over to fill in a self-report questionnaire after first excluding patients who had a life expectancy less than three months, suffered from dementia, did not understand Dutch, planned to move or spend a long time abroad, or lived in a nursing home.[32] Participants who were at increased risk of functional decline based on a score of two or more on the Identification of Seniors At Risk—Primary Care (ISAR-PC) were eligible for study participation.[33] The ISAR-PC was developed to identify community-living older persons at increased risk of functional decline and is validated in Dutch. It comprises three dichotomous risk factors for functional decline, has a moderate predictive value, and is easy to use.[33]

### Randomization

We performed a computerized stratified cluster randomization procedure, in which practices were the clusters. We stratified on socio-economic status, number of enlisted patients, and practitioners in both study groups.[31]

### Intervention

The participants from practices randomized to the intervention group received nurse-led multifactorial care provided by the community-care registered nurses (CCRN) in addition to the usual care provided by the GP. The intervention was designed to identify and treat geriatric problems in an early stage, and to improve care coordination between healthcare professionals.[31, 32] The program included a comprehensive geriatric assessment (CGA), an individually tailored care and treatment plan (CTP) consisting of multifactorial interventions, and nurse-led care coordination with multiple follow-up home visits. The CGA focused on somatic, psychological, functional and social domains, including a physical examination and performance tests to identify conditions such as urinary incontinence, memory problems, increased risk of falling, and loneliness.[31, 32] Possible interventions were referral to a GP, referral to a paramedic, giving advice, or follow-up visit by the CCRN.[34]

### Care as usual

The participants from general practices randomized to the usual care group received no extra care or information besides usual care. In the Dutch healthcare system, the general practitioner (GP) plays a central role as the gatekeeper of the healthcare system, (s)he is the first and only freely accessible medical professional, and people are used to visiting their GP first if they have a health problem.[35]

### Outcome measurements

All data were collected using self-report questionnaires at baseline, six and twelve months. The primary outcome was participants’ change in (I)ADL measured with the modified Katz-ADL index score at one year of follow-up.[36] The modified Katz-ADL index score ranges from zero to 15 points with higher scores indicating more dependence in (I)ADL. The secondary outcome included Quality-Adjusted Life-Years (QALYs) based on the EuroQol (EQ-5D-3L) over one year.[37] The EQ-5D-3L is a five-dimension scale to measure health related quality of life. Resulting health states were converted to utilities using the Dutch EQ-5D-3L tariff which was based on a sample of the Dutch general population.[38] The utilities reflect the relative desirability of a particular health state where 0 indicates death and 1 indicates perfect health. We calculated QALYs by multiplying the utilities by the amount of time spent in a particular health state. Changes in health states between measurements were considered to be linear.

### Healthcare utilization and costs

Healthcare utilization and associated costs were measured from a healthcare perspective. During one year, healthcare utilization rates were collected using self-report questionnaires at baseline, six and twelve months to measure the number of GP consultations, the number of GP visits after hours, the hours of personal care and home nursing received, the number of days receiving day care and residential care, the number of nursing home admission days, the number of emergency room visits, and the number of hospital admissions. The EMR of participants admitted to one hospital (n = 1421) were used to determine the mean length of stay per hospital admission at six and twelve months per participant. This data was subsequently used to calculate the total number of hospital admission days. Dutch standard costs were used to value healthcare utilization according to Dutch guidelines for costing research.[39] All prices were adjusted for the year 2016 using consumer price index figures.[40] Cost were calculated by multiplying the volumes of healthcare utilization with cost prices of that unit. S1 Table lists the cost categories and prices used in the CEA and the CUA.

To calculate the costs of the intervention, time to identify older persons at increased risk and time to train nurses for the intervention was valued using salary costs (S2 Table). In addition, the CCRNs used diaries to fill in the number of visits, and time per visit for each participant including consultation time with the GP. A top down approach was used by calculating the total costs of intervention (including the people screened) and dividing this by the total number of participants in the intervention group.

### Statistical analysis

All analyses were performed according to the intention-to-treat principle. Baseline characteristics of participants were described for the two study groups. Missing values for cost and effect data were imputed using multiple imputation by chained equations (MICE) with predictive mean matching (PMM).[41, 42] Individual sub costs per category were imputed instead of total costs to maximize the accuracy of the imputation.[43] We created 50 imputed datasets that were analyzed separately. The analysis results from the imputed datasets were pooled using Rubin’s rules.[44]

Seemingly unrelated regression analyses were performed to estimate adjusted cost and effect differences. Cost and effect differences were adjusted for confounding variables, which were selected on the basis of causal diagrams. Confounders included baseline values of (i) the outcome variable, (ii) age, (iii) sex, (iv) education (three categories), and (v) socio-economic status (three categories).[45] Incremental Cost-Effectiveness Ratios (ICERs) were calculated by dividing the pooled cost difference by the pooled effect difference. Bias-corrected accelerated bootstrapping techniques (5000 replications) were used to estimate 95% confidence intervals around cost and effect differences, and the statistical uncertainty around the ICER. The uncertainty surrounding the ICER was visually presented in the cost-effectiveness (CE) planes.

Cost-effectiveness acceptability curves (CEAC) were estimated to quantify the uncertainty due to sampling and measurement errors. The CEAC is a plot of the probability that the intervention is cost-effective in comparison with care as usual (y-axis) as a function of the money society might be willing to pay for one additional unit of outcome (x-axis). The pooled coefficients and variance parameters from the regression models were used to estimate the CEACs.[46]

We used IBM SPSS, Version 20.0 (IBM Corp. 2011) and STATA 13 (StataCorp. 2013. College Station, TX) for data analysis.

## Results

**Fig 1:**
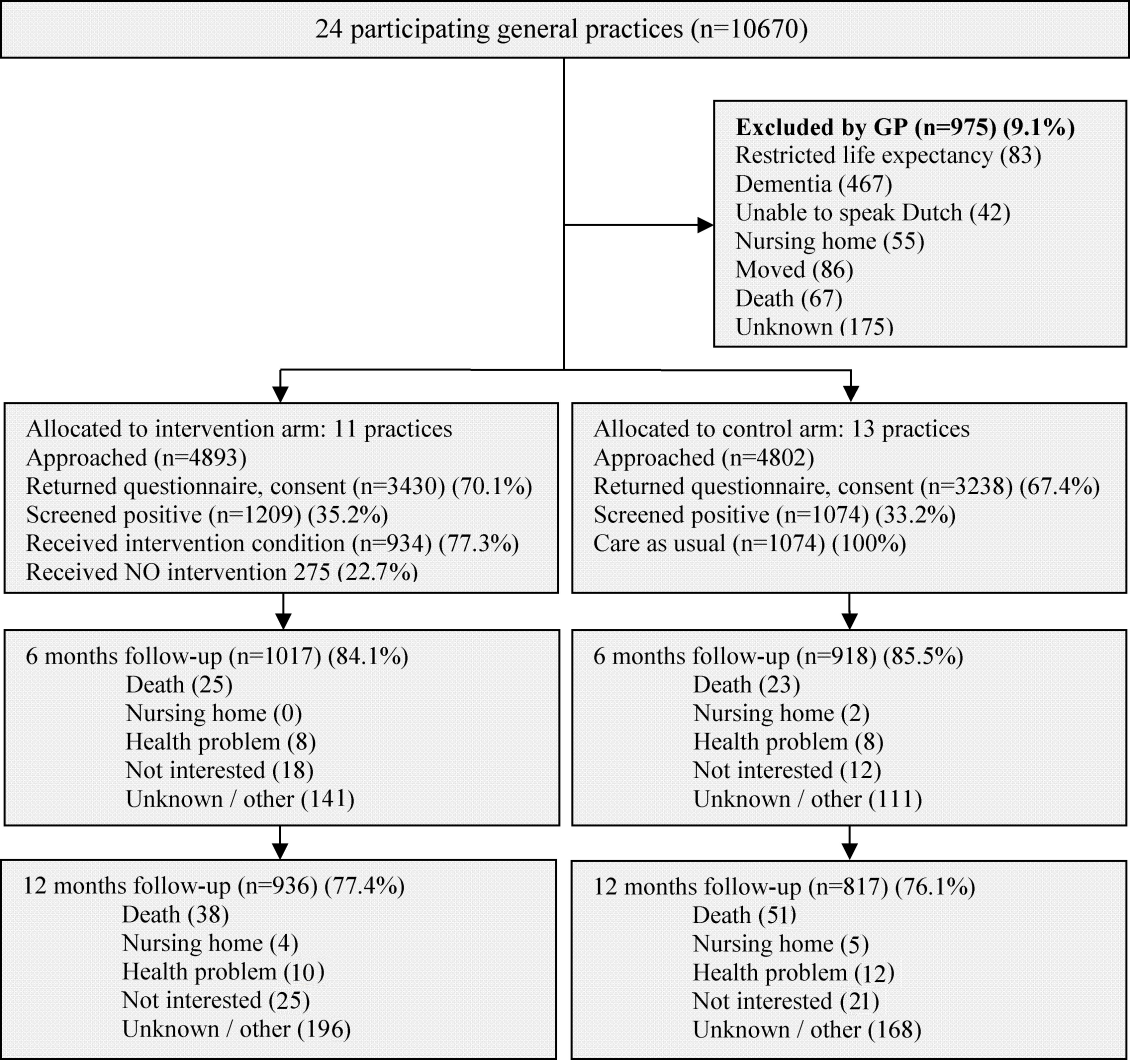
Flow of practices and participants through the trial. Eleven practices were randomized to the intervention group and 13 practices were randomized to the usual care group. In both groups around 35% of the invited persons were at increased risk of functional decline and participated in the study. In both groups the follow-up rates were around 77% after one year respectively.

Eleven practices were randomized to the intervention group and 13 practices were randomized to the usual care group. In total, 35.2% (1209/3430) of the potential participants in the intervention group and 33.2% (1074/3238) of the potential participants in the usual care group (Fig 1) were at increased risk of functional decline based on a score ≥ 2 points on the ISAR-PC. The participants’ baseline characteristics were balanced between both study groups, except that there was a higher percentage of people with a high SES level in the intervention group (Table 1). The median age of the participants was almost 83 years in both groups. The median modified Katz-ADL index score was 2 (IQR 1–5) points in the intervention group and 3 (IQR 1–5) points in the usual care group. The follow-up rates after one year were 77.4% (936/1209) in the intervention group and 76.1% (817/1074) in the usual care group (Fig 1). At one year of follow-up, 23% of the cost data were missing, 24% of the modified Katz-ADL index data were missing and 23% of the QALY data were missing. The participants who declined the comprehensive geriatric assessment (n = 275) were older, had more (I)ADL disabilities, and more often lived in a home for the elderly (data not shown).

**Table 1:** Distribution of baseline variables of participants with an ISAR-PC score ≥ 2, by study group (N = 2283).

Characteristics		Intervention group	Usual care group
		N = 1209	N = 1074
		n(%)	n(%)
Age, in years, median (IQR)		82.6 (76.8–86.8)	82.9 (77.3–87.3)
Female sex		789 (65.2)	671 (62.7)
Caucasian		1141 (95.4)	1022 (96.5)
Level of education			
	primary school or less	255 (21.3)	281 (26.6)
	secondary education	760 (63.7)	648 (61.4)
	college or university	179 (15.0)	127 (12.0)
Socio-economic status			
	low (≤1SD)	57 (4.7)	78 (7.3)
	intermediate	931 (76.9)	890 (83.2)
	high (≥1SD)	223 (18.4)	102 (9.5)
Married/living together		561 (46.7)	489 (46.0)
Living situation			
	independent	530 (44.0)	467 (43.9)
	aloneindependent	535 (44.5)	442 (41.6)
	togetherhome for elderly	138 (11.5)	154 (14.5)
Multimorbidity (≥2)		997 (83.2)	856 (80.6)
Polypharmacy (≥3)		830 (69.3)	748 (70.7)
Modified Katz-ADL index (range 0–15), (median (IQR))		2 (1–5)	3 (1–5)
Katz-ADL (range 0–6), median (IQR)		1 (0–1)	1 (0–1)
IADL scale (range 0–7), median (IQR)		1 (0–3)	2 (0–3)
EuroQol-5D (range -0.33–1.0), mean (SD)		0.75 (0.21)	0.72 (0.22)
Emotional wellbeing (Rand-36) (range 4–100), mean (SD)		71.4 (17.4)	70.3 (17.6)
Self-perceived quality of Life (scale range 0–10), mean (SD)		7.2 (1.3)	7.2 (1.2)
Healthcare utilization in past 12 months			
	hospital admission (≥1)	306 (26.1)	264 (25.6)
	GP after hours (≥1)	232 (20.1)	175 (17.2)
	home nursing	193 (17.0)	149 (14.7)
	personal care	654 (56.3)	523 (51.9)
	day care	26 (2.2)	36 (3.5)
Falls (≥1) in past 12 months		418 (34.9)	344 (32.7)
Identification of seniors at risk-primary care (range 0–7.5), median (IQR)		4 (3–5)	4 (3–5)

Values are numbers (percentages) unless stated otherwise. IQR = interquartile range; SD = standard deviation

### Healthcare use and costs

The mean total costs after one year were EUR 6518 (SE 472) per participant in the intervention group and EUR 5214 (SE 338) in the usual care group (Table 2). The costs for the intervention were EUR 156 per participant. The mean total costs for GP care per participant were significantly lower in the intervention group (adjusted mean difference EUR -13 (95% CI: -21; -4). Mean cost for personal care and nursing home admission per participant were significantly higher in the intervention group as compared to the usual care group (adjusted mean difference personal care: EUR 247 (95% CI: 60; 453); nursing home: EUR 520 (95% CI: 189; 968)). Mean total home care costs and long-term care contributed most to the adjusted mean differences in total cost between both groups. The adjusted mean difference in total costs between the intervention and the usual care group was €1457 (95% CI: 572; 2537) indicating significantly higher cost in the intervention group.

**Table 2:** Mean costs in intervention and usual care group after one year.

		Intervention group	Usual caregroup	unadjusted mean difference in costs
		N = 1209	N = 1074	EUR
		mean costs (Standard Error)^a^^e^	mean costs (Standard Error)^a^^e^	(95% Confidence Interval)^b^
GP care				
	GP consult	71 (2)	79 (3)	**-8 (-15; -1)**
	GP after hours	15 (2)	22 (2)	**-5 (-11; 0)**
	Total GP care	86 (3)	100 (4)	**-14 (-23; -4)**
Home care				
	Home nursing	2239 (316)	1873 (231)	366 (-280; 1169)
	Personal care	2383 (85)	2118 (77)	**266 (65; 487)**
	Total Home care	4621 (356)	3991 (261)	630 (-105; 1535)
Long-term care				
	Daycare	23 (4)	23 (4)	1 (-9; 10)
	Elderly home	428 (100)	552 (116)	-124 (-352; 111)
	Nursing home	909 (242)	350 (106)	**559 (203; 1041)**
	Total long-term care	1361 (312)	924 (180)	437 (-70; 1071)
Secondary care				
	Emergency room	37 (3)	44 (4)	-6 (-17; 2)
	Hospital	676 (98)	552 (81)	124 (-53; 306)
	Total secondary care	712 (99)	595 (83)	126 (-65; 302)
Intervention (nurse patient, nurse GP, screening)		168	0	
Total costs		7012 (508)	5609 (364)	**1338 (332; 2514)**

^a^ Mean cost per participant were unadjusted

^b^ 95% confidence intervals were estimated using bootstrapped bivariate regression models ^b^ Bold = significant difference

^e^ Dutch standard costs were used to value healthcare utilization. All prices were adjusted for the year 2016 using consumer price index figures

### Outcomes of disability and QALYs

The adjusted effect of the intervention after one year was -0.07 (95% CI -0.22 to 0.07) on the modified Katz-ADL index and 0.004 (95% CI -0.009–0.017) on the QALY indicating no significant differences in clinical outcomes between groups.

### Cost-effectiveness analysis

**Fig 2:**
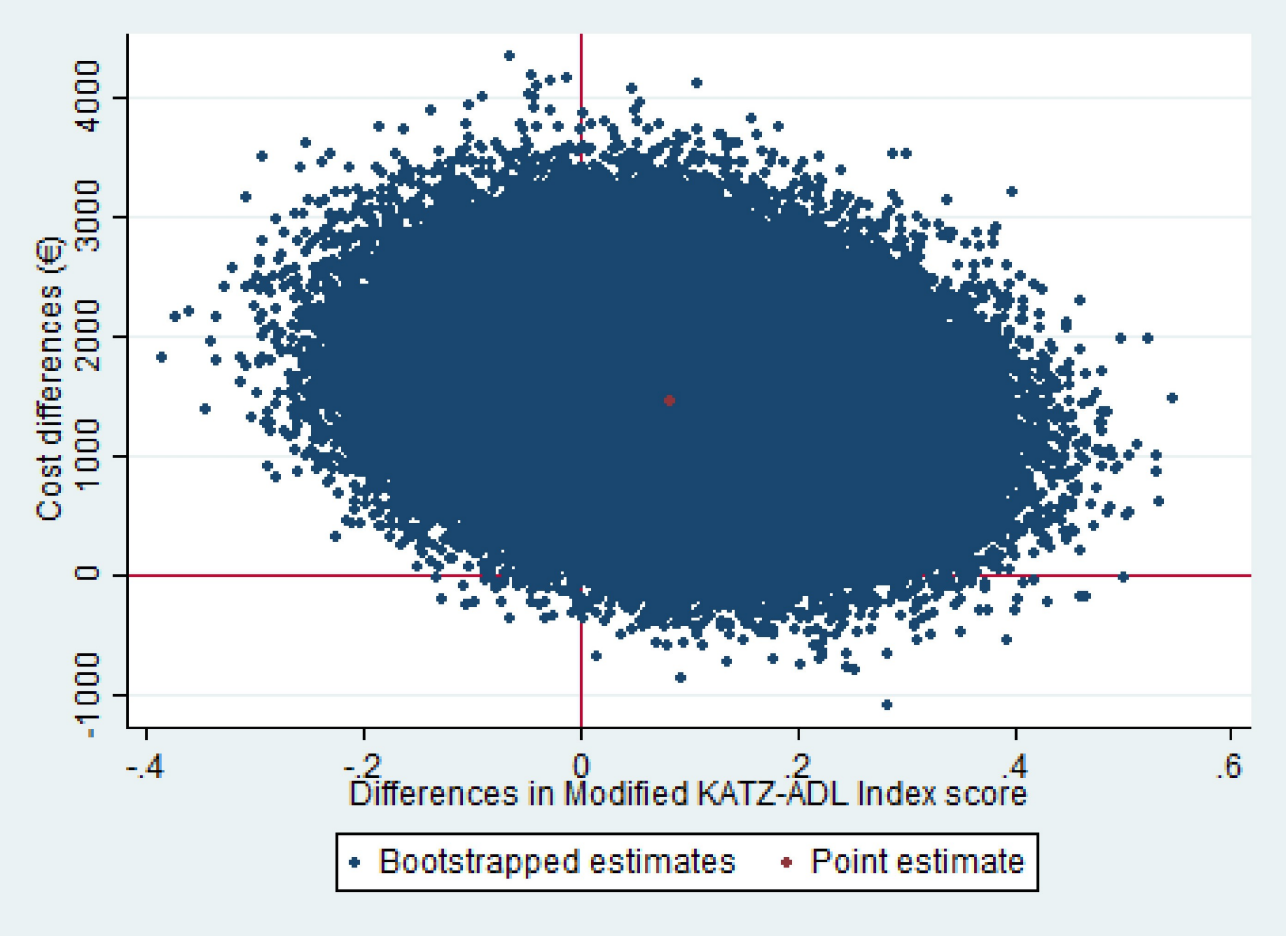
Cost-Effectiveness (CE) Planes for disability. The cost-effectiveness (CE) planes visualize the uncertainty surrounding the ICER estimated using bootstrapped bivariate regression models while adjusting for confounders. Modified Katz-Activities of daily living index score: (range 0–15). The effect difference was multiplied by -1 to keep the CE plane understandable (northeast: more effective, more expensive; southeast: more effective, less expensive; southwest: less effective, less expensive; northwest: less effective, more expensive).

**Fig 3:**
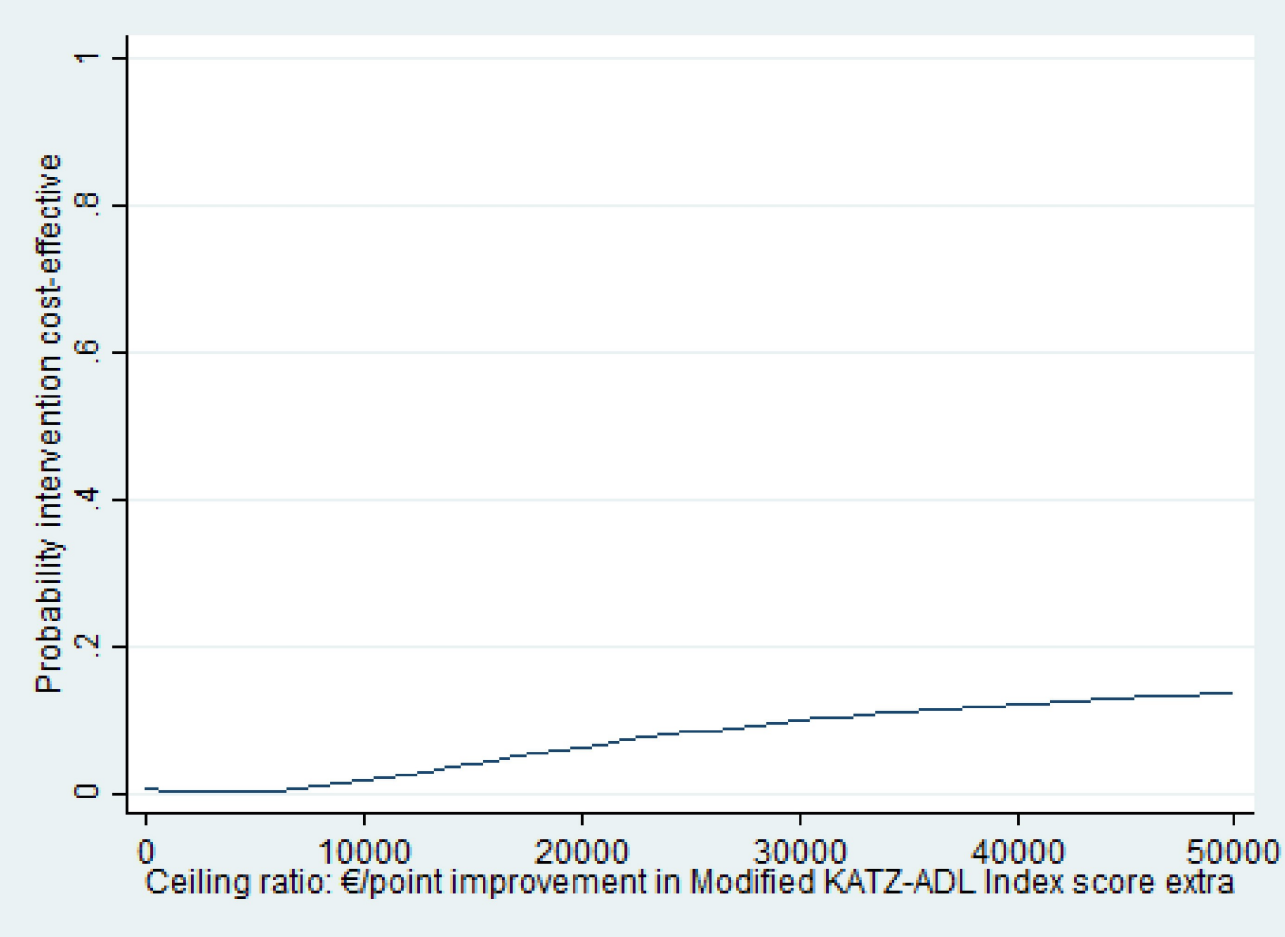
Cost-effectiveness acceptability curves (CEAC) for disability. The CEAC is a plot of the probability that the intervention is cost-effective in comparison with usual care (y-axis) as a function of the money society might be willing to pay for one additional unit of outcome (x-axis) Modified Katz-Activities of daily living index score

The ICER for the modified Katz-ADL index was 21,884 meaning that to gain one point of improvement in modified Katz-ADL index EUR 21,884 needs to be invested in the intervention group as compared to usual care. Fig 2 presents the cost-effectiveness plane for the modified Katz-ADL index. The majority (79%) of the cost-effect pairs for the modified Katz-ADL index were in the northeast quadrant indicating that the intervention was on average more effective and more expensive than usual care. The cost-effectiveness acceptability curves (CEAC) for improvement in modified Katz-ADL index score is shown in Fig 3. The CEAC for the modified Katz-ADL index shows that the probability that the intervention group was cost-effective in comparison with the usual care group was less than .01 at a willingness to pay (WTP) of 0 EUR/point improvement in modified Katz-ADL index score and this increased to 0.14 at a WTP of 50,000 EUR/point improvement in modified Katz-ADL index score.

### Cost-utility analysis

**Fig 4:**
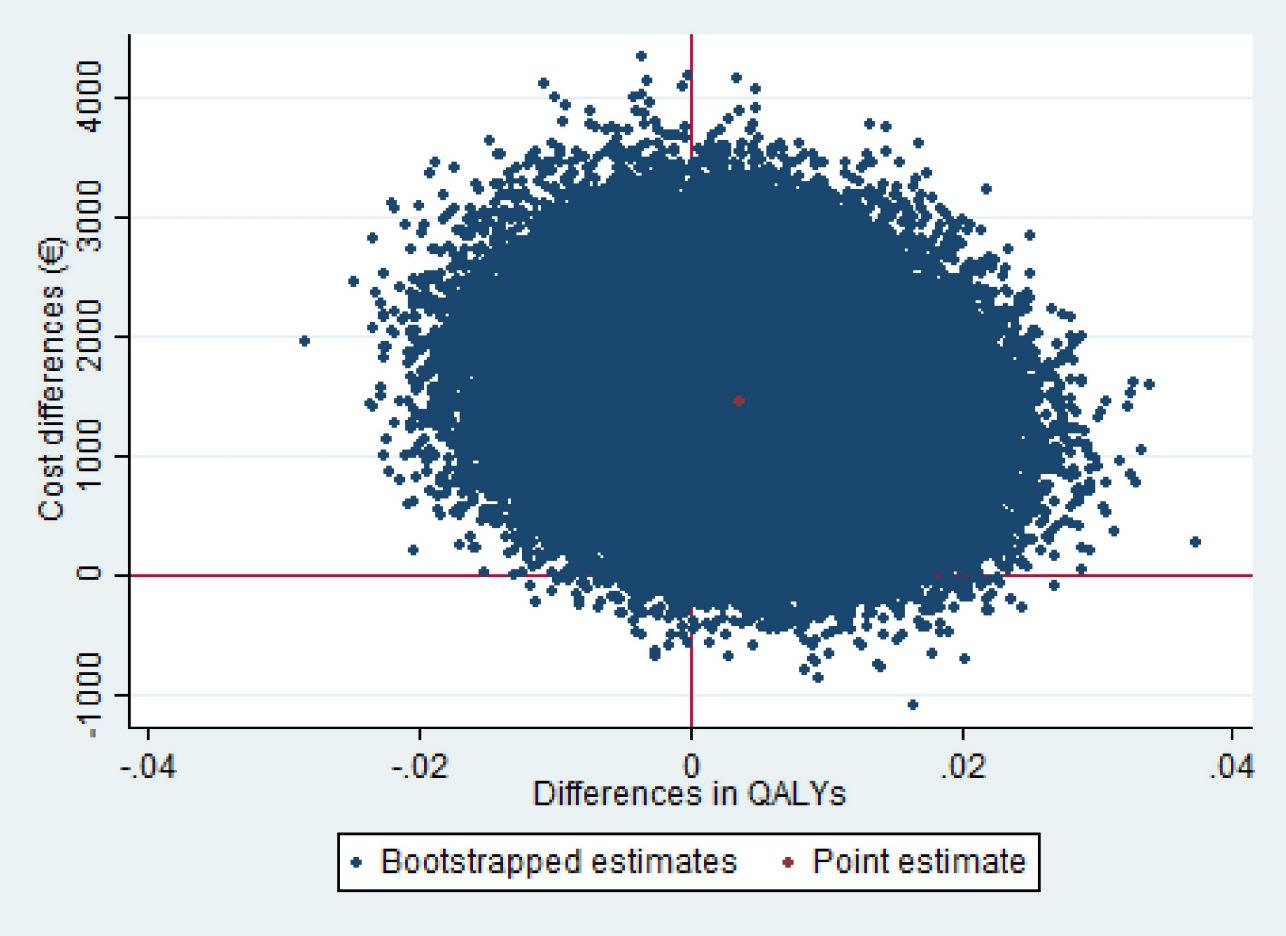
Cost-Effectiveness (CE) Planes for QALY. Qaly = Quality-adjusted-life-year: (range -0.33–1.0) positive results indicate improvement in QALY

**Fig 5:**
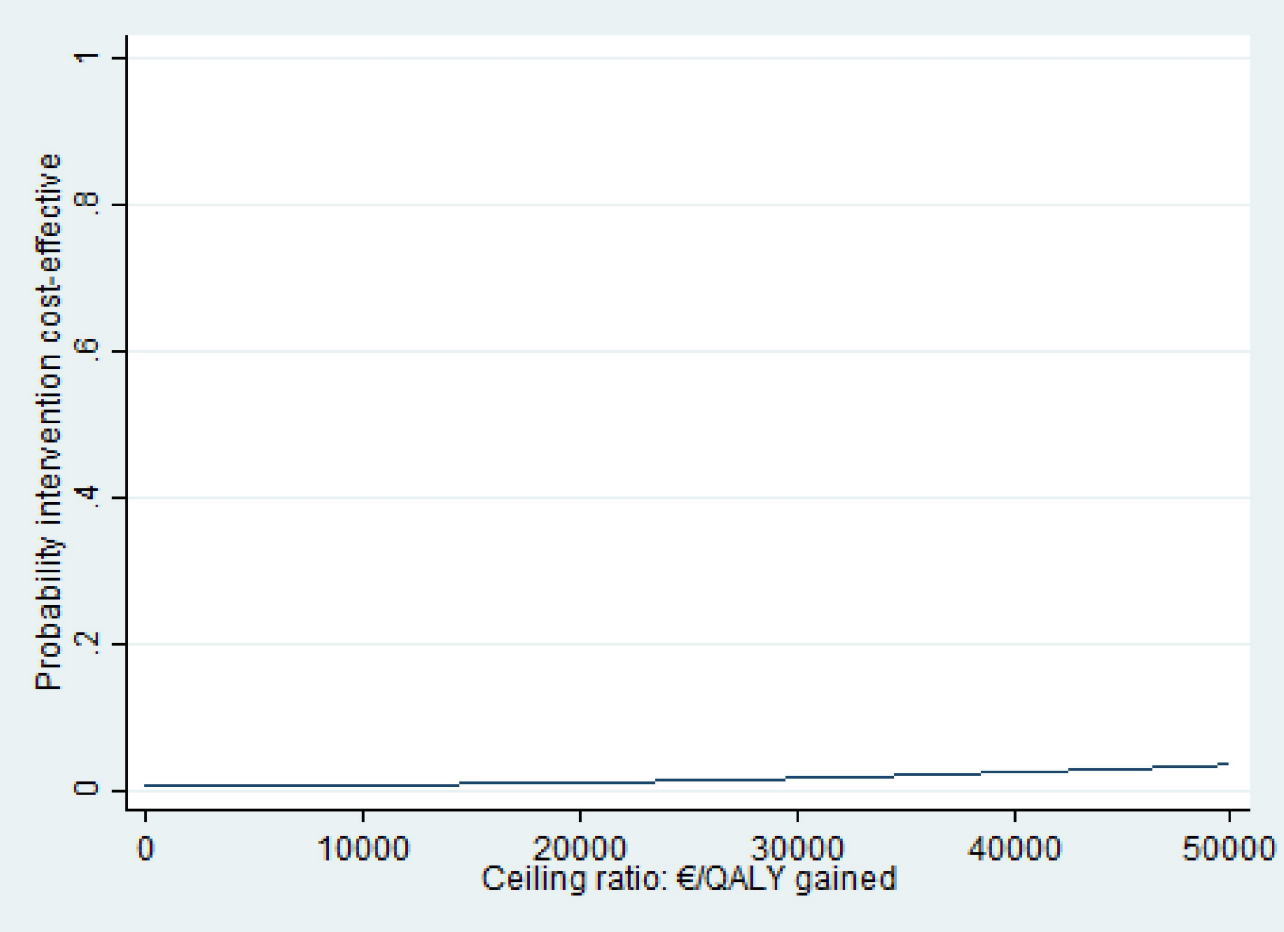
Cost-effectiveness acceptability curves (CEAC) for disability. Qaly = Quality-adjusted-life-year

The ICER for QALYs was 287,879 indicating that to gain one QALY an additional EUR 287,879 needs to be invested in the intervention group as compared with usual care. For QALYs, the majority of the cost-effect pairs was also situated in the northeast quadrant of the CE plane (Fig 4). The probability of cost-effectiveness at a WTP of 0 EUR/ QALY gained was less than .01 as well, and this increased to 0.04 at a WTP of 50,000 EUR/QALY gained (Fig 5).

## Discussion

The results of our study show that a one-year nurse-led multifactorial care program is not cost-effective compared with care as usual in community-living older people at increased risk of functional decline in the Netherlands. There were no statistically significant differences in Katz-ADL index score and QALYs between the two groups. Costs in the intervention group were significantly higher than in the usual care group. Costs for personal care and nursing home nursing were significantly higher in the intervention group as compared to the usual care group.

### Strengths and limitations

Our study has several strengths. We conducted a large cluster randomized trial in a population at increased risk of functional decline. Other strengths of the study include the patient-centered approach, the high participation rate, the high adherence rate to the structured study protocol, and the evidence-based toolkit. Some limitations should also be taken in consideration. First, the cost-effectiveness analysis was conducted from a healthcare perspective instead of societal perspective which is recommended in to Dutch guidelines for costing research.[39] Lost productivity costs were not included because these costs are not considered relevant at this age. Informal care data were collected, but could not be used because of a considerable amount of missing data due to non-response. It is possible that when more appropriate care is arranged after discharge the amount of informal care needed is reduced resulting in lower informal care costs in the intervention group as compared to the usual care group. For this study, we focused on healthcare services that contribute most to total costs (home care, long-term care, and secondary care). However, since the intervention did not specifically target hospital outpatient care, medication use, or consultations with allied healthcare professions (physiotherapy, occupational therapy), these services were not included. In total, 14% (254/1856) of all initiated interventions consisted of referrals to allied health care professionals and 7% (136/1856) of all interventions concerned medication use. Not including allied healthcare professions and medication use may have resulted in an underestimation of the intervention cost and total societal costs in the intervention group. However, since these costs only represent a small proportion of the total costs, we expect that it is unlikely that including these costs would have changed the overall conclusion of this study. Second, healthcare utilization was measured using self-report questionnaires. As people become older and more functionally impaired, collecting healthcare utilization from the EMR should be considered to avoid measurement bias.[47] However, no uniform source is available to collect all healthcare utilization. To increase the validity of the largest cost driver, hospital admissions, we used data from the hospital EMR to calculate these costs. Third, in the intervention group at one year of follow-up around 23% of total cost and 24% of the effect data were missing. To prevent bias, we imputed missing values for cost and effect data using multiple imputation with predictive mean matching (PMM). Multiple imputation is highly preferred above naïve imputation methods such as Last Value Carried Forward (LVCF) which has been shown to bias parameter estimates.[41].

### Comparison with other studies

Before the start of this study, cost-effectiveness analyses on multifactorial care to prevent or postpone new disabilities were scarce.[19] Recent studies in the Netherlands and Finland which were performed from a societal perspective, found no evidence for the cost-effectiveness of multifactorial preventive interventions in preventing or postponing new disabilities as compared with usual care.[13–16, 48] This is consistent with the results of our study. Two other recent studies which were performed from a societal perspective, found that their intervention was cost-effective at high willingness to pay ratios (EUR 20,000 and 50,000 Australian dollars (= EUR 32,327) respectively).[17, 18] These positive results can largely be explained by fewer hospital admissions and nursing home admissions, and less informal care in the intervention groups as compared to the control groups resulting in cost savings.[17] However, considering the heterogeneity in the study populations and interventions in these different studies, it is difficult to adequately explain these contradicting results. A large variety consists between the total cost per participant over a one-year period between different studies in the intervention group (the estimated total amounts vary between EUR 13,251 and EUR 19,353).[15, 48] The total cost per participant described in our study are substantially lower than the total costs described in these other studies. This may have resulted from the exclusion of informal care cost in our study. However, home care and nursing home admission were the main cost drivers in other studies as well.[15, 18, 48]

### Explanation of findings and implications for future research

There are several possible explanations why we found no evidence of cost-effectiveness in our study. First, the cost-effectiveness analyses were conducted after one year, which may have been too short to see preventive effects emerge. When targeting a pre-frail population, and aiming to prevent future incidents, longer intervention and follow-up periods may be necessary to be able to show beneficial effects on new disabilities, hospital admission and costs.[11, 48–50] Higher costs for personal care and home nursing admission in our study may have been the direct result of the initiated interventions. This ‘intervention effect’ has been described before.[15, 16, 48, 51, 52] Second, the effect estimate of the intervention on disability (-0.07 on the modified Katz-ADL index) and on health-related quality of life (0.004 on the EQ-5D) was very small and not clinically relevant. The outcome measure, the modified Katz-ADL index may not be sensitive enough to detect clinically relevant change in disability over time and may poorly discriminate between people with relatively few disabilities in a community-dwelling setting.[53, 54] Besides, the EQ-5D-3L that was used to measure health-related quality of life strongly focusses on health, whereas in older adults aspects of quality of life beyond health, may be more important contributors to quality of life in older people. The Adult Social Care Outcomes Toolkit (ASCOT) is an example of an outcome measure that assesses quality of life from a broader perspective than health alone, and that can be used in economic evaluations of care interventions for older people.[55]

### Conclusions and implications for further research

In this cost-effectiveness analysis, we found that one-year multifactorial nurse-led care was not cost-effective compared to usual care in community-living older people at increased risk of functional decline in The Netherlands. This adds to the accumulating evidence that proactive integrated care provision over a one-year period is not (cost-)effective in preventing new disabilities in countries with a well-developed primary care system. Based on these findings, implementation of the evaluated multifactorial nurse-led care model is not to be recommended. However, the implementation of multifactorial nurse-led care in general practice is ongoing in many healthcare systems throughout the Western world. In view of the ageing of Western societies, and the burden the care for older people incurs for GPs, increasing task delegation from GPs to nurses warrant further evaluation with regard to both quality of life and costs. Such studies should indicate whether programs like these may result in valuable service provision at acceptable costs and quality.

## Supporting information

S1 Table Healthcare utilization and costs. (DOCX)

S2 Table Intervention costs. (DOCX)
