# Supplementary material for: Cost-effectiveness of nurse-led multifactorial care to prevent or postpone new disabilities in community-living older people: Results of a cluster randomized trial
Source: PLoS One. 2017 Apr 17;12(4):e0175272. doi: 10.1371/journal.pone.0175272 (PMC5393862; doi:10.1371/journal.pone.0175272)
Supplement: S1 Table — (DOCX) [file pone.0175272.s001.docx]

**S1 Table. Healthcare utilization and prices (euro, 2016) used in this study**

| **Healthcare utilization** | | **Volume** | **Prices** |
| --- | --- | --- | --- |
|  |  |  | (euro, 2016) |
| General practitioner (GP) | |  |  |
|  | GP Consultation | Number | 31.2 |
|  | GP Consultation after hours | Number | 62.0 |
|  | |  |  |
| Home care | |  |  |
|  | Home nursing | Hour | 53.9 |
|  | Personal care | Hour | 26.8 |
|  | |  |  |
| Long term care | |  |  |
|  | Daycare | Day | 111.9 |
|  | Residential care | Day | 100.4 |
|  | Nursing home | Day | 265.3 |
|  | |  |  |
| Secondary care | |  |  |
|  | Emergency room | Number | 168.4 |
|  | Hospital admission | Day | 484.9 |

Prices are obtained from the Dutch manual for cost-analysis in healthcare research (2010). Subsequently, prices per categories were indexed to the reference year (2016) using a consumer price index.
