# Supplementary material for: Cost-effectiveness of nurse-led multifactorial care to prevent or postpone new disabilities in community-living older people: Results of a cluster randomized trial
Source: PLoS One. 2017 Apr 17;12(4):e0175272. doi: 10.1371/journal.pone.0175272 (PMC5393862; doi:10.1371/journal.pone.0175272)
Supplement: S2 Table — (DOCX) [file pone.0175272.s002.docx]

**S2 Table. Intervention costs**

| **Intervention costs** | **Minutes**) | **Costs** | **Total costs** | **Cost per participant**) |
| --- | --- | --- | --- | --- |
|  | (per participant | (euro 2016, per hour) | (euro) | (n=1209 |
| **Intervention** |  |  |  |  |
| Postal screening | - | - | 6129.6 | 5.1 |
| Training | - | - | 6502.9 | 5.4 |
| Nurse | 120 | 64.5 | 156069.7 | 129.1 |
| GP-nurse consultation | 10 | 169.4 | 34139.9 | 28.3 |
|  |  | | |  |

Prices are obtained from the Dutch manual for cost-analysis in healthcare research (2010). Subsequently, prices per categories were indexed to the reference year (2016) using a consumer price inde
